# Anti-angiogenic therapies for the treatment of angiosarcoma: a clinical update

**DOI:** 10.1007/s12254-017-0365-x

**Published:** 2017-11-07

**Authors:** Robin J. Young, Penella J. Woll

**Affiliations:** Academic Unit of Clinical Oncology, Weston Park Hospital, Whitham Rd, S10 2SJ Sheffield, United Kingdom

**Keywords:** Angiosarcoma, Sarcoma, Anti-angiogenic therapy

## Abstract

Angiosarcomas are rare aggressive endothelial tumours, and are associated with a poor prognosis. Due to their vascular nature, there is great interest in their response to anti-angiogenic agents. A number of small prospective studies have reported angiosarcoma response to vascular-targeted agents, including agents that target vascular endothelial growth factor. To date, the response to these agents has been disappointing, and similar to the response observed in other soft tissue sarcoma subtypes. This short review will summarise the recent data in this field.

## Introduction

Angiosarcomas are rare aggressive endothelial tumours [1]. Angiosarcomas can occur spontaneously or secondary to radiotherapy, chronic lymphoedema and in a variety of familial conditions including neurofibromatosis (NF-1), mutated breast cancer associated gene 1 or 2 (BRCA1 or BRCA2) and Maffucci syndrome. They can develop at any site, but most commonly present as cutaneous disease, either as primary tumours on the head and neck of elderly white men, or on the chest wall secondary to breast cancer treatment. Complete excision with wide margins is often technically challenging due to disease extent and infiltration of surrounding tissue, effects of previous surgery/radiotherapy on local anatomy including lymphoedema, and co-morbidities in older patients. Angiosarcomas frequently recur despite surgery. Doxorubicin or paclitaxel are chemotherapies recommended for the treatment of recurrent/advanced disease. Evaluation of new agents has been hampered by the rarity of angiosarcomas, but also by the difficulty in assessing response in diffuse infiltrating tumours. Hence progression-free survival (PFS) and overall survival (OS) are more robust endpoints than response rate.

Over the past 20 years, the tumour vasculature has become established as a target in the treatment of cancer, particularly with agents that target vascular endothelial growth factor (VEGF). As angiosarcomas are endothelial tumours, there is interest in the response of angiosarcoma to vascular-targeted agents. This short review will summarise the recent data in this field.

## Paclitaxel

Paclitaxel is a microtubule-targeting chemotherapy with anti-angiogenic activity [2]. Response to weekly paclitaxel (80 mg/m^2^) was assessed in a prospective single arm phase II study of 30 patients with advanced angiosarcoma [3]; 36% of patients had received previous chemotherapy. The response rate (complete response [CR] + partial response [PR]) was 19%. Three patients with locally advanced disease had tumour responses that enabled subsequent surgery, and pathological CR were reported in 2 cases. The median PFS was 4 months, and the median OS was 7.4 months. Angiosarcoma responses to doxorubicin and paclitaxel have not been directly compared, but these results are similar to those obtained with anthracycline-based chemotherapy in 108 angiosarcoma patients prospectively enrolled in European Organisation for the Research and Treatment of Cancer (EORTC) trials, where the response rate was 25%, with median PFS and OS of 4.9 and 9.9 months respectively, which were not significantly different to those in other soft tissue sarcomas (STS) [4]. Weekly paclitaxel is well tolerated and is often favoured as first-line therapy, particularly for the treatment of cutaneous angiosarcoma and in elderly patients.

## Agents targeting VEGF

### Bevacizumab

Bevacizumab is a humanised monoclonal antibody to VEGF-A. Angiosarcoma response to bevacizumab monotherapy (15 mg/kg 3 weekly) was studied in a single arm phase II study, which also recruited patients with epithelioid haemangioendothelioma [5]. In all, 23 patients with advanced angiosarcoma were recruited, and in these patients, the response rate was 9% (2/23); 43% (10/23) of patients progressed within 6 weeks, and the median PFS was 3 months.

The addition of bevacizumab (10 mg/kg 2 weekly) to weekly paclitaxel (90 mg/m^2^) was studied in a non-comparative, open-label, randomised phase II study [6]. The response rate was lower with combination therapy (29% [7/25] *vs* 46% [11/24]), but the 6 month PFS rate was identical. Furthermore, the addition of bevacizumab to paclitaxel was associated with higher rates of serious toxicity (44% *vs* 22%), and more patients receiving combination treatment required dose reductions (56% *vs* 35%). The addition of bevacizumab to weekly paclitaxel is therefore not recommended.

### Oral tyrosine kinase inhibitors

Angiosarcoma response to oral tyrosine kinase inhibitors that target VEGF receptors (VEGFR) has been studied in several phase II studies.

Tumour response to sorafenib (a VEGFR, Raf-1, and B‑Raf inhibitor) 400 mg bis die (bd) was reported in a study of 122 advanced sarcoma patients stratified by histological subtype [7]. One CR and 4 PR were observed in 34 patients with angiosarcoma. The median PFS of the vascular sarcoma cohort, which included 2 patients with solitary fibrous tumour and 1 patient with giant haemangioma, was 3.8 months. In comparison, the median PFS across all sarcoma subtypes was 3.2 months. Angiosarcoma response to sorafenib 400 mg bd was studied by the French Sarcoma Group in two patient cohorts: 26 patients with cutaneous angiosarcoma and 15 patients with visceral disease [8]. The primary endpoint for this study was the 9‑month progression-free rate. Only 2 patients, 1 from each cohort, were progression free at 9 months. The median PFS was 1.8 and 3.8 months for cutaneous and visceral disease respectively.

STS response to pazopanib (a VEGFR, fibroblast growth factor receptor [FGFR] and platelet derived growth factor receptor [PDGFR] inhibitor) 800 mg omni die (od) was studied in the phase II EORTC 62043 [9], and subsequent randomised placebo-controlled phase III EORTC 62072 (PALETTE) study [10]. The median PFS of patients that received pazopanib in the PALETTE study was 4.6 months, compared to 1.6 months in patients that received placebo (HR 0.31, 95% CI 0.24–0.40; *p* < 0.0001). Angiosarcoma response to pazopanib was not reported separately; however a retrospective analysis of a cohort of 40 angiosarcoma patients treated with pazopanib in either the EORTC 62043 study, the PALETTE study, or in routine clinical practice, reported a 20% response rate and median PFS of 3.0 months [11]. There were no significant differences observed between different subtypes of angiosarcoma in response to pazopanib.

Angiosarcoma response to axitinib (a VEGFR, PDGFR and KIT inhibitor) 5 mg bd was recently studied within a UK histologically stratified phase II study (NCT01140737), which also included cohorts for patients with advanced leiomyosarcoma, synovial sarcoma and ‘other’ STS histologies. Preliminary results from the angiosarcoma cohort were reported at the Connective Tissue Oncology Society 2016 annual meeting; the response rate was 10%, 12 week non-progression rate 42%, and median PFS 4.2 months [12].

## Agents targeting non-VEGF angiogenic pathways

### Angiopoietin 1/2

Angiosarcoma response to weekly trebananib (30 mg/kg), an angiopoietin-1 and -2 peptibody, was studied in a small single arm phase II study. No responses were observed and the median PFS was 1.6 months. STS response to regorafenib 160 mg od, a multi-kinase inhibitor targeting VEGFR, PDGFR, Kit, RET and Raf-1, as well as TIE2, an angiopoietin receptor, was studied in a histologically stratified, randomised, placebo-controlled phase II study [13]. Similar to STS response to pazopanib, an unplanned pooled analysis of non-adipocytic STS cohorts reported a median PFS 4.0 months with regorafenib compared to 1.0 months with placebo (HR 0.36, 95% CI 0.25–0.53; *p* < 0.0001). Angiosarcoma response to regorafenib was not reported, but a separate study of regorafenib for advanced angiosarcoma is currently ongoing (NCT02048722).

### PDGF

In addition to VEGFR, pazopanib, regorafenib and axitinib also inhibit PDGFR. Olaratumab is a humanised monoclonal antibody to PDGFa. Promising results were observed in a randomised phase I/II study of olaratumab in combination with doxorubicin as first-line treatment for advanced STS [14]; a phase III study recently completed recruitment and results are eagerly awaited (NCT 02451943). Angiosarcoma response to olaratumab has not been reported.

### Endoglin

The transforming growth factor (TGF)-β and bone morphogenetic protein (BMP)-9 receptors activin receptor-line kinase (ALK)-1 and endoglin are required for normal vascular development, and are highly expressed on tumour endothelial cells [15]. TRC105 is a chimeric monoclonal antibody that binds endoglin [16]. Non-adipocytic STS response to TRC105 in combination with pazopanib was studied in a phase I/II study [17]; response to treatment was observed in 8/9 angiosarcoma patients, including two durable CR. A randomised phase III trial of pazopanib ± TRC105 in angiosarcoma recently opened to recruitment (NCT02979899).

## Other vascular targeted agents

### Thalidomide

Angiosarcoma response to thalidomide, an immunomodulatory agent with anti-angiogenic properties [18], has been described in several case reports [19, 20], however response has not been studied in a prospective trial.

### Metronomic chemotherapy

Metronomic chemotherapy is the concept of administering chemotherapy drugs in frequent, often daily, low doses. It reduces tumour micro-vessel density, tumour hypoxia, and improves tumour perfusion, consistent with a vascular-targeted response [21]. Angiosarcoma response to metronomic chemotherapy has been reported in a few case studies [22]. A retrospective series of 26 elderly patients with advanced STS treated with metronomic oral cyclophosphamide 100 mg bd, which included 3 patients with angiosarcoma, reported a disease control rate of 69% and a median PFS of 6.8 months [23].

### Beta-blockers

The beta-blocker propranolol is recommended for the treatment of complex infantile haemangiomas [24]. Interestingly, beta-adrenergic receptors have also been identified as a potential therapeutic target in malignant vascular tumours [25]. A case series of 7 patients with advanced angiosarcoma treated with oral propranolol 40 mg bd in combination with weekly metronomic vinblastine (6 mg/m^2^) plus methotrexate (35 mg/m^2^) reported a 100% response rate and a median PFS of 11 months [26].

## Discussion

Despite their vascular nature, angiosarcoma response to VEGF-targeted agents is limited, and similar to the response observed in other more common STS subtypes. Biomarkers are required to identify patients most likely to respond to treatment. In a small cohort study, activating VEGFR2 mutations were identified in 14% (3/22) of angiosarcoma tumour samples [27]. No VEGFR2 mutations were identified in 27 tumour samples available for analysis from the French Sarcoma Group study of sorafenib for advanced angiosarcoma [8]. In a separate study, mutations in angiogenesis-related genes were identified in 38% (15/39) of angiosarcoma tumour samples [28], including recurrent mutations in vascular endothelial-phosphotyrosine phosphatase (VE-PTP) and phospholipase gamma 1 (PLCG1). The response of angiosarcomas harbouring mutations in angiogenesis-related genes to vascular targeted agents has not been correlated.

Angiosarcoma tumour cells express multiple angiogenic growth factors [29]. Angiosarcoma expression of these factors is associated with improved patient outcomes [30], and thus expression may be a measure of tumour endothelial differentiation rather than a pathological driver of disease. Furthermore, the expression of multiple angiogenic growth factors represents potential resistance mechanisms to targeted agents. Despite the negative results of paclitaxel in combination with bevacizumab [6], further combination studies—either anti-angiogenic agents in combination with chemotherapy or combinations of different anti-angiogenic agents—are indicated.
